# Plasmonic-tape-attached multilayered MoS_2_ film for near-infrared photodetection

**DOI:** 10.1038/s41598-020-68127-7

**Published:** 2020-07-09

**Authors:** Minji Park, Gumin Kang, Hyungduk Ko

**Affiliations:** Nanophotonics Research Center, Korea Institute of Science and Technology, Hwarangno 14-gil 5, Seongbuk-gu, Seoul, 02792 South Korea

**Keywords:** Engineering, Materials science, Nanoscience and technology, Optics and photonics

## Abstract

Molybdenum disulfide has been intensively studied as a promising material for photodetector applications because of its excellent electrical and optical properties. We report a multilayer MoS_2_ film attached with a plasmonic tape for near-infrared (NIR) detection. MoS_2_ flakes are chemically exfoliated and transferred onto a polymer substrate, and silver nanoparticles (AgNPs) dewetted thermally on a substrate are transferred onto a Scotch tape. The Scotch tape with AgNPs is attached directly and simply onto the MoS_2_ flakes. Consequently, the NIR photoresponse of the MoS_2_ device is critically enhanced. The proposed tape transfer method enables the formation of plasmonic structures on arbitrary substrates, such as a polymer substrate, without requiring a high-temperature process. The performance of AgNPs-MoS_2_ photodetectors is approximately four times higher than that of bare MoS_2_ devices.

## Introduction

Two-dimensional (2D) transition metal dichalcogenides (TMDCs) show excellent optoelectronic performance because their bandgap can be adjusted by controlling the thickness. This can be advantageous for flexible optoelectronic devices on an atomic scale^1–5^. Among TMDCs, molybdenum disulfide has attracted considerable attention in optoelectronic applications such as light-emitting devices and photodetectors because of its high transparency, high carrier mobility, and mechanical flexibility^6–9^. However, MoS_2_ is typically employed for detecting wavelengths in the visible range, and its detection performance in the near-infrared (NIR) range is limited due to its intrinsic band structure. For MoS_2_ films synthesized using chemical exfoliation^10–12^, the interlayers with van der Waals bonding in a bulk-layered material are broken and intercalated with small ions such as lithium^12^. Chemically exfoliated MoS_2_ films can partially absorb infrared light because the MoS_2_ flakes chemically exfoliated using organolithium have a high content of metallic 1 T phase MoS_2_^10–14^. It was reported that chemically exfoliated MoS_2_ films extended their absorption up to the wavelength of 1,550 nm in the NIR region.

It is well known that plasmonic nanostructures can strongly induce a localized near-field. Various low-dimensional photodetectors combined with plasmonic nanostructures have demonstrated excellent device performance^15–23^. Previously, we reported that chemically exfoliated MoS_2_ could generate photocurrent by extending NIR light absorption up to the wavelength of 1,550 nm, and that the responsivity could be improved by simply forming a plasmonic nanostructure as an underlayer of MoS_2_^24^. That is, a Ag thin film was deposited and annealed to form randomized silver nanoparticles (AgNPs) successively on a substrate, and finally, a MoS_2_ film was layered on the AgNPs array. However, this thermal dewetting method is not applicable to polymer substrates, and the surface roughness due to the metal nanoparticles (NPs) may cause unexpected electrical and mechanical problems in the active layer of MoS_2_ flakes.

In this paper, we introduce a plasmonic-tape-attached multilayered MoS_2_ device to enhance NIR absorption and consequently photocurrents. In this device, chemically exfoliated MoS_2_ is first transferred onto a substrate, and then a plasmonic tape is directly attached to the MoS_2_ film through a Scotch tape. Herein, a plasmonic tape refers to a composite film of transparent Scotch tape and embedded metal NPs at the adhesive surface. The plasmonic tape can be reproducibly fabricated by chemically treating the surface of a substrate onto which a metal thin film is deposited, and then removing it with Scotch tape. The plasmonic tape can be reproducibly fabricated using Scotch tape by taping and peeling thermally dewetted metal NPs from the chemically treated surface of a substrate. Therefore, this plasmonic tape is suitable for thermally or chemically weak substrates that cannot directly form plasmonic NPs via thermal annealing or chemical etching. In addition, the tape film itself serves as a passivation layer that can protect a device from moisture penetration or mechanical damage without deteriorating the electrical and optical properties. We systematically investigate the optoelectrical properties and photodetection performance of the plasmonic MoS_2_ device at the NIR wavelengths of 980 and 1,550 nm. We report that the plasmonic MoS_2_ device yields a sensitivity approximately four times that of the bare MoS_2_ device.

## Results

**Figure 1:**
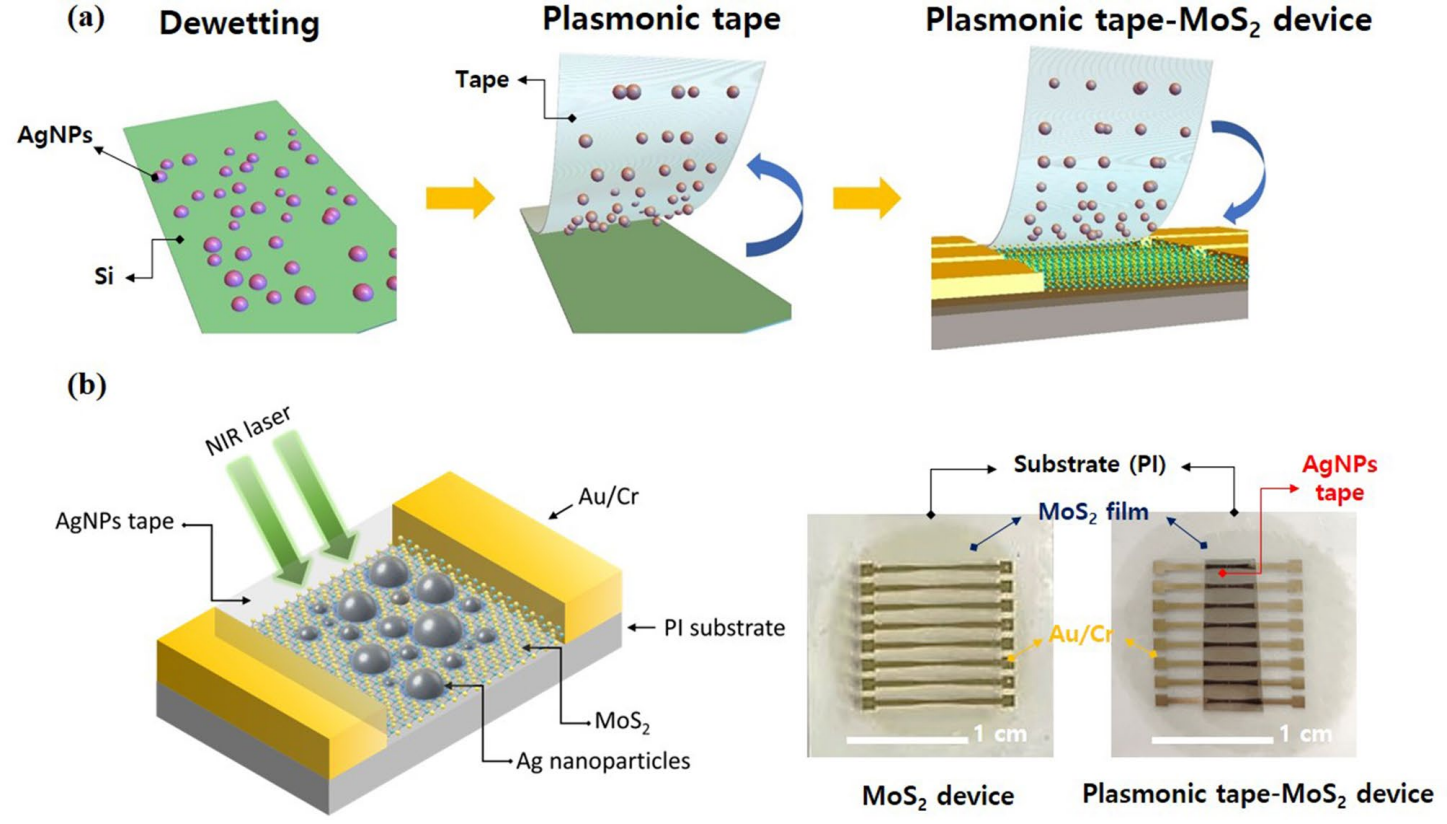
(**a**) Fabrication process of the MoS_2_ photodetectors decorated with plasmonic AgNPs. Thermally dewetted AgNPs were detached from Si substrate using a 3 M tape and attached to the surface of a MoS_2_ device. (**b**) Schematic illustration and photograph of the plasmonic-tape-MoS_2_ photodetector.

**Figure 2:**
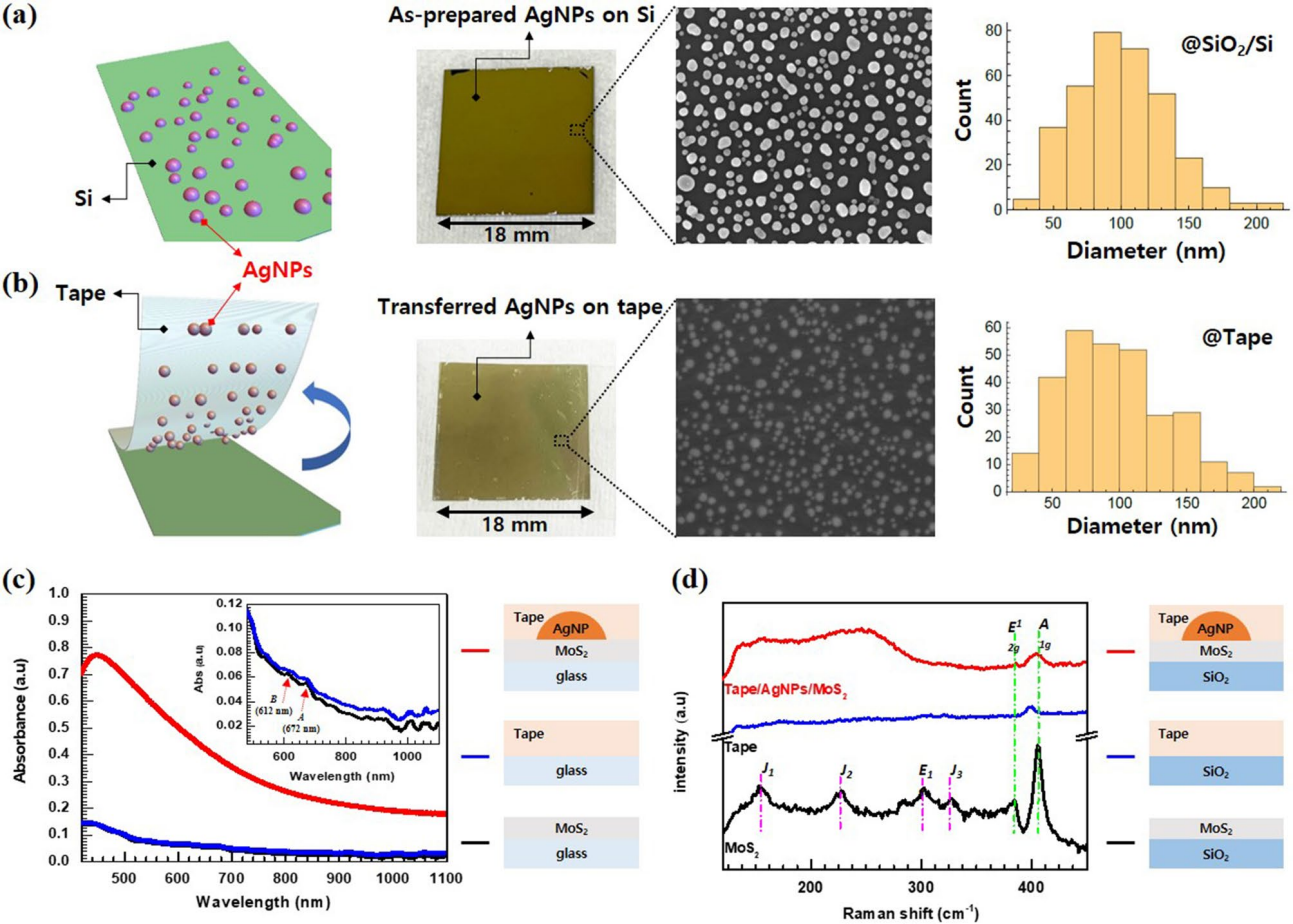
Photographs, SEM images, and size distribution histograms of (**a**) as-prepared AgNPs on Si substrate and (**b**) AgNPs transferred onto 3 M tape. (**c**) UV–Vis–NIR absorbance spectra of multilayered MoS_2_ on a glass substrate with and without plasmonic AgNPs. (**d**) Raman spectra of plasmonic tape/MoS_2_, bare tape, and bare MoS_2_ film on the glass substrate.

The schematic fabrication process of the MoS_2_ device attached with the plasmonic AgNPs-tape film is briefly illustrated in Fig. 1a and the detailed procedure are shown and described in Figures S1–S3 and “Methods”. Figure 1b shows the photographs of the samples of the bare MoS_2_ device and the plasmonic-tape-attached MoS_2_ device. Figure 2a,b show the photographs, scanning electron microscopy (SEM) images and size distribution histograms of AgNPs formed on the Si substrate and transferred onto the 3 M tape (i.e., plasmonic tape film), respectively. The AgNPs are randomly distributed and are elliptical or circular in shape. The average diameters of the bare and transferred AgNPs are ~ 100.85 nm and ~ 96.82 nm, respectively. The size of the AgNPs before and after the tape transfer is believed to be almost the same, even though the transferred AgNPs are likely to appear small due to the low contrast ratio caused by the polymer adhesive. Moreover, the transfer rate of the AgNPs from Figure S4 was estimated to be ~ 99% according to atomic force microscopy (AFM) analysis. Therefore, the transfer process was very effective without serious loss of AgNPs. A Cary 5,000 UV–VIS-NIR spectrometer was used to study the optical properties of the bare MoS_2_ and plasmonic-tape-MoS_2_ (i.e., AgNPs/MoS_2_) films. Multilayered MoS_2_ films were transferred onto glass substrates to measure absorption spectra. In Fig. 2c, the AgNPs/MoS_2_ film shows absorption enhancement over a broad band of spectra at all wavelengths, including the visible range; thus, the MoS_2_ film becomes less transparent upon attaching the plasmonic tape. In the inset of Fig. 2c, the bare MoS_2_ film exhibits peaks A and B at 672 nm and 612 nm, respectively, corresponding to the two MoS_2_ direct band gap transitions^25–27^, whereas it shows slightly increased absorption and a broad absorption tail, which indicate the indirect band transition. In the NIR (900–1,600 nm) region, the AgNPs/MoS_2_ sample exhibits nearly four times stronger absorption than the bare MoS_2_ film, owing to the plasmonic NIR absorption in the structure. Raman measurements were obtained using a Renishaw (inVia Raman Microscope) spectrometer with an excitation wavelength of 532 nm. Figure 2d shows the Raman spectra of the multilayered MoS_2_ without and with the plasmonic tape, where a bare Scotch-tape-attached sample is used for comparison. The Raman spectrum of multilayered MoS_2_ film displays an in-plane active mode *E*_*2g*_^*1*^ at ~ 384 cm^−1^ and an out-of-plane mode *A*_*1g*_ at 405 cm^−1^. In addition, it exhibits additional peaks, 156 (*J*_*1*_), 226 (*J*_*2*_), and 333 (*J*_*3*_) cm^−1^, featuring 1 T-MoS_2_^28–30^. After attaching the plasmonic tape on top of MoS_2_, an overall increase in the intensity of the Raman spectra is observed. Especially, for the out-of-plane *A*_*1g*_ modes, additional peaks appeared and became broader because of the plasmonic effect of AgNPs. However, other peaks did not appear due to the tape.

**Figure 3:**
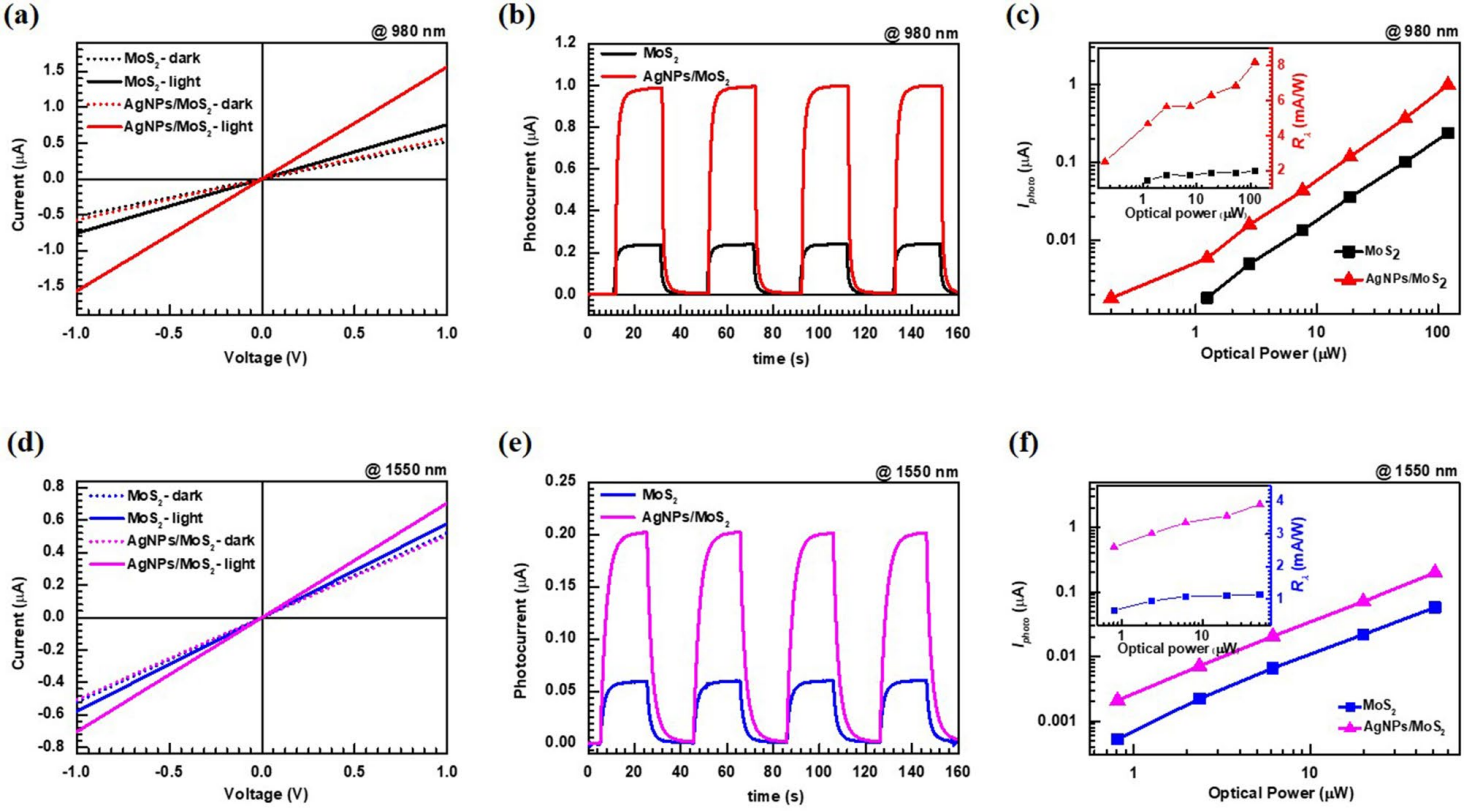
(**a**) *I–V* characteristics of the MoS_2_ photodetectors. (**b**) Transient photocurrent of the photodetectors at V_DS_ = 1 V, and (**c**) photocurrent with respect to illumination power of the devices at the wavelength of 980 nm. The inset shows the responsivity as a function of illumination light power. (**d**–**f**) The data acquired similarly at the wavelength of 1,550 nm.

Figure 3a shows the *I*_*ds*_*–V*_*ds*_ curves of bare MoS_2_ and plasmonic-tape-MoS_2_ devices under the illumination of wavelength 980 nm at 121.03 µW where the channel length and width are 20 µm and 200 µm, respectively. The curves show good linearity, indicating that ohmic contacts between the MoS_2_ films and the electrodes are well formed. The curves for the plasmonic samples show the same distinct linear characteristics as those of the bare MoS_2_ films, indicating that the attached AgNPs do not affect the interfacial electrical properties of the MoS_2_ layer. The plasmonic AgNPs-MoS_2_ photodetectors show an evident improvement of photocurrent at 980 nm compared with the bare MoS_2_ photodetectors. As shown in Fig. 3b, the transient photoresponses of the MoS_2_ photodetectors without and with AgNPs are characterized using a light pulse at *V*_*DS*_ = 1 V under a wavelength of 980 nm at 120 µW. We confirm that the device exhibits stable and repeatable switching characteristics under NIR laser irradiation at 980 nm. Figure 3c shows the output characteristics of the photocurrents of the devices based on ${I}_{ph}={I}_{illumination}-{I}_{dark}$ without and with the plasmonic film. It can be observed that the photocurrents of the plasmonic-MoS_2_ photodetector are four times higher than those of the MoS_2_ device. The illumination power dependence shows that the photocurrent increases linearly with the illumination power, for the cases without and with AgNPs. The external responsivity ${(R}_{\lambda })$ and detectivity $({D}^{*})$ of the bare and plasmonic MoS_2_ photodetectors are defined as ${R}_{\lambda }= \frac{{I}_{ph}}{{P}_{Light}}$ and ${D}^{*}= \frac{{R}_{\lambda }}{{(2q{I}_{dark}/A)}^{1/2}}$, respectively^6,8^, where ${I}_{ph}={I}_{illumination}-{I}_{dark}$ is the photocurrent, *P*_*Light*_ is the power of the incident light applied to the channel, *A* is the active area of the detector, *q* is the absolute value of an electron charge ($1.6 \times {10}^{-19}$ C), *R*_*λ*_ is the responsivity measured in units of *AW*^*−1*^, and D* is the detectivity measured in units of Jones.

Thus, the responsivity of the plasmonic device is enhanced by approximately four times compared with that of the bare device within the optical power range of 1–120 µW shown in Fig. 3c. Specifically, at *V*_*DS*_ = 1 V, the responsivity values are ~ $8\times {10}^{-3}A{W}^{-1}$ and ~ 2 $\times \;{10}^{-3}A{W}^{-1}$ at optical power of 100 µW for the plasmonic and bare devices, respectively. At illumination intensities lower than 1 µW (e.g., at 0.2 µW), the AgNPs-MoS_2_ photodetector showed photoresponsivities of $\sim 3.1\times {10}^{-3}A{W}^{-1}$, but the bare MoS_2_ photodetector did not exhibit a photoresponse. The corresponding ${D}^{*}$ value of the plasmonic device (i.e., $\sim 1.2\times {10}^{6}$ Jones) is increased by ~ 3.8 times with respect to that of the bare device (i.e., $\sim 3.1\times {10}^{5}$ Jones). The enhancement of the ${D}^{*}$ value is slightly smaller than the enhancement of the *R* value because the dark current increases slightly for the plasmonic device.

Similarly, we performed the above characterization for the devices without and with the plasmonic AgNPs tape under a wavelength of 1,550 nm. From Fig. 3d, it is observed that all the current–voltage curves are linear and the obtained photocurrents of the AgNPs-MoS_2_ photodetectors are significantly enhanced under the same power illumination compared with that of the bare MoS_2_ photodetectors. As shown in Fig. 3e, we also confirmed the reproducible time-resolved photoresponse of the devices at 1,550 nm. The photocurrent has good linearity with the irradiation powers in the measured range in Fig. 3f. Consequently, the *R* and D* values of the plasmonic device are enhanced by 3.5 and 3.3 times, respectively, with respect to those of the bare device, where the *R* values are $\sim 1.1\times {10}^{-3}A{W}^{-1}$, $\sim 3.9\times {10}^{-3}A{W}^{-1}$, and the D* values are $\sim 1.7\times {10}^{5}$ Jones, $\sim 5.8\times {10}^{5}$ Jones, for the bare and plasmonic devices, respectively at the optical power of 50 µW. And, comparative I*–*V curves of the MoS_2_ photodetectors under the illumination of different wavelengths are provided in Figure S5. Therefore, it was confirmed that the plasmonic tape strongly contributed to the increase in the MoS_2_ photocurrent, by effectively absorbing NIR radiation not only at 980 nm but also at 1,550 nm. The device exhibited robust, stable, and repeatable characteristics. And, the previously reported photodetectors based on plasmonic-2D materials are summarized in Table S1. Unlike the previous reports, our device was fabricated by a 2D material thin film of centimeter scale by chemical exfoliation method, and it was possible to fabricate the device with a simple shadow mask process without complicated lithography process, and improve the device performance by post-processing of the plasmonic tape.

**Figure 4:**
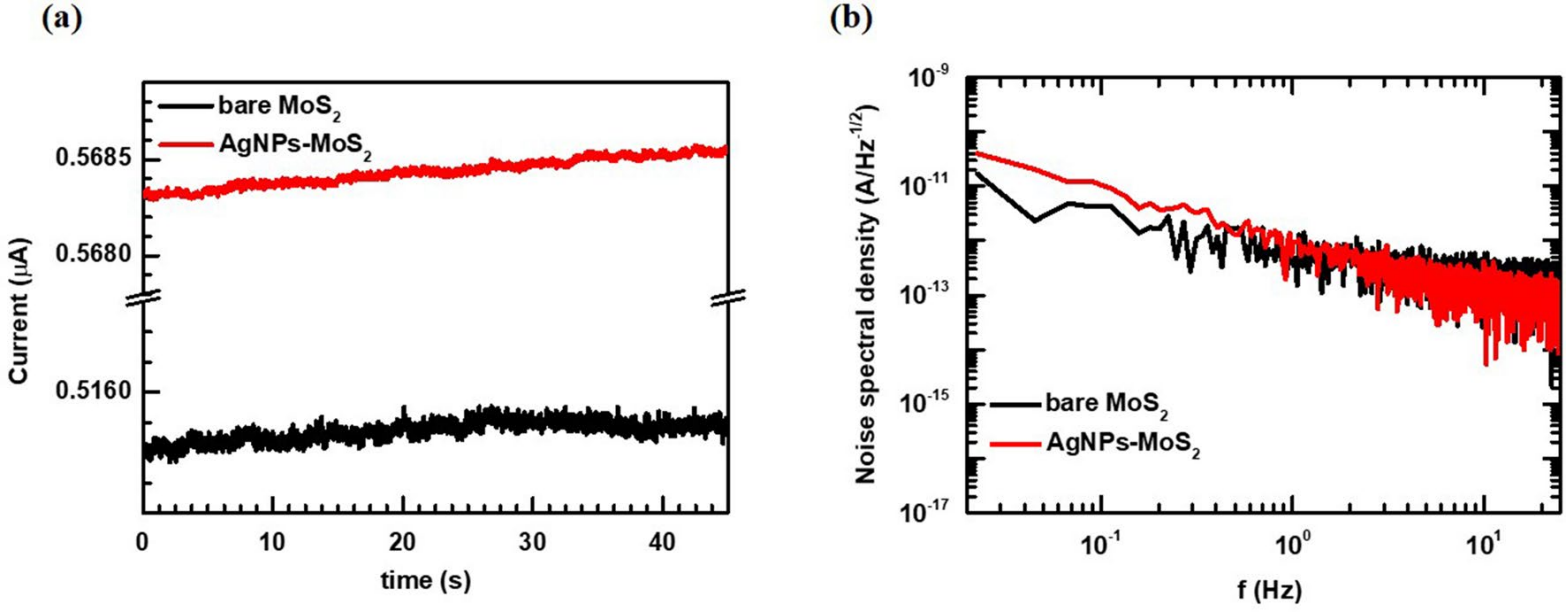
(**a**) Dark current waveform of the bare and plasmonic MoS_2_ photodetectors. (**b**) Analysis of the noise spectral density of the bare and plasmonic MoS_2_ photodetectors.

In addition, we measured the dark current of both devices and analyzed its noise spectral density. As shown in Fig. 4, the dark current of the device did not critically increase with the introduction of the plasmonic tape. Furthermore, in the analysis of the noise spectral density obtained using the fast Fourier transform (FFT) of the dark currents^31^, the plasmonic-tape-MoS_2_ device exhibits similar noise level to the bare MoS_2_ device. This indicates that the attached plasmonic tape does not affect the low-frequency noise characteristic of the bare device, which follows the 1/*f* noise theory. Although the metal NPs decorated on an active layer may possibly result in an increase of the dark current in the device, the introduction of our plasmonic tape does not cause a deterioration of such electrical characteristics.

**Figure 5:**
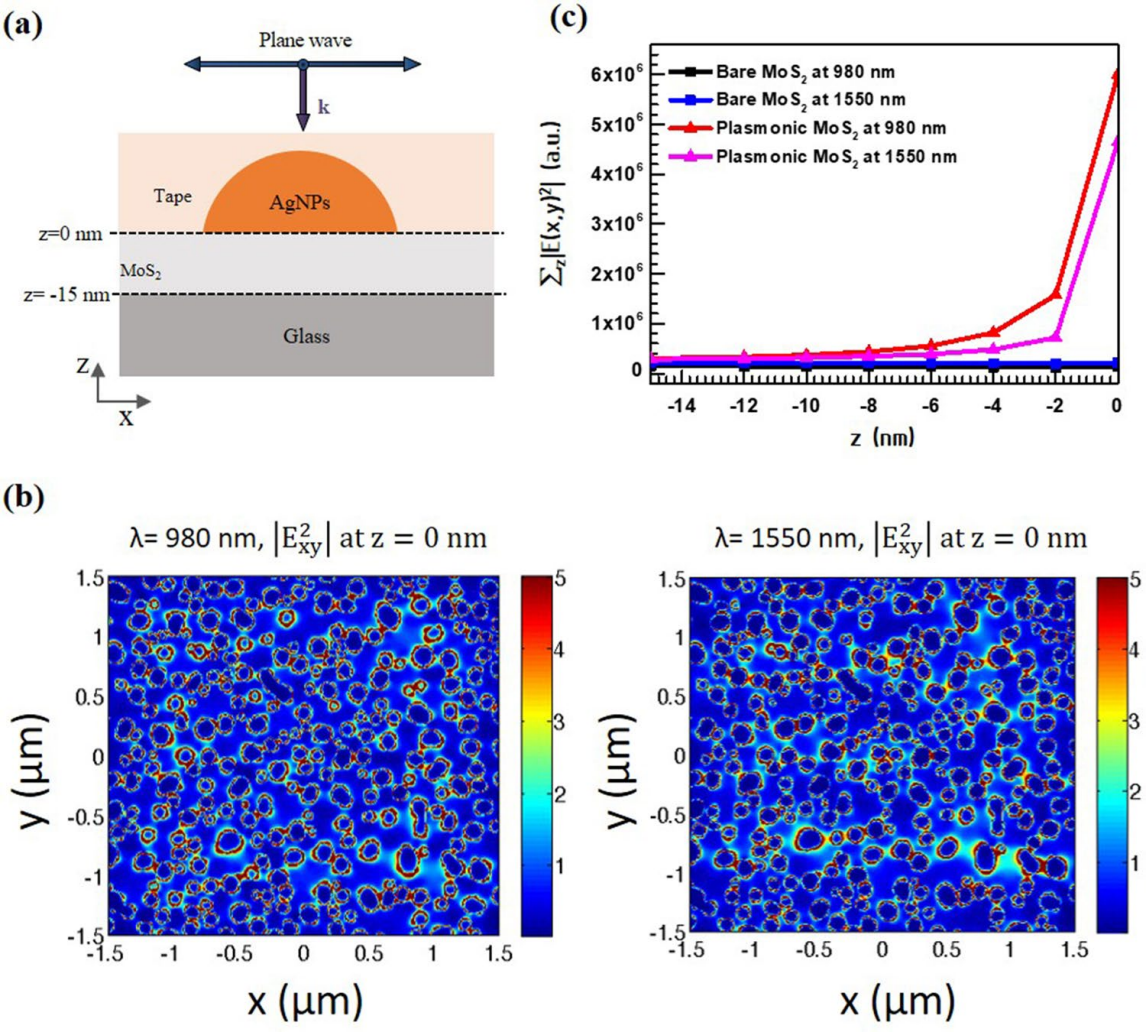
(**a**) Schematic illustration of the modeling geometry used in the FDTD numerical simulation. (**b**) E-field intensity profiles in the *XY* plane at the interface between AgNPs and MoS_2_ (i.e., *z* = 0 nm) when the wavelength of the incident light is 980 nm and 1,550 nm. (**c**) A plot of the integrated electromagnetic field intensity in the *XY* plane along the *z*-axis (∑_z_|E(x,y)^2^|).

We employed a three-dimensional finite-difference time-domain (FDTD) simulation for the dimensions of 3 × 3 μm^2^ in the *XY* plane in Fig. 5 to analyze the effect of the plasmonic tape. For the 3D simulation, an unpolarized plane wave source is applied normally to the plane in the backward direction with the boundary condition of perfectly matched layer (PML). The *E*-field intensity, (|*E*_un_|^2^), is obtained by averaging the *x*- (|*E*_x-pol_|^2^) and *y*-polarized (|*E*_y-pol_|^2^) profiles (i.e., |*E*_un_|^2^ = 1/2(|*E*_*x*-pol_|^2^ +|*E*_*y*-pol_|^2^)). The complex refractive index of MoS_2_ used for simulation is approximately extracted from a literature^32^ and the index is 4 + 0.01*i* for the NIR wavelengths.

The plasmonic structure was modeled using Wolfram Mathematica (ver. 11.1.1.0) based on the SEM image in Fig. 2a. In the plasmonic-tape-MoS_2_ configuration, a strongly localized near-field in the proximity of the AgNPs is observed (Fig. 5b), mainly resulting from the excitation of the localized surface plasmon (LSP) modes. The plasmoic tape-MoS_2_ exhibits broadband light absorption including the visible and NIR wavelengths, as shown in the simulated absorption spectra of Figure S6. The randomly arranged AgNPs array in the plasmonic tape functions as a scattering center for broadband wavelength as well as a nano-antenna inducing the localization of E-filed due to the LSPs from an individual AgNP and interparticle interaction among AgNPs. The randomly distributed AgNPs array with various particle sizes and spacing extends the LSP excitation wavelength up to NIR range. Thus, the plasmonic-tape-MoS_2_ configuration can meaningfully absorb the incident NIR radiation because of the LSP mode generated around the AgNPs. Specifically, at both 980 nm and 1,550 nm, strong hot spots are observed at the interface between MoS_2_ and AgNPs, enhancing the integrated values of the E-field intensity at the interface (i.e., *z* = 0) as much as 45.5- and 21.6-fold for the wavelengths 980 nm and 1,550 nm, respectively, compared with those of the bare MoS_2_, in Fig. 5c. As expected, the strongest increase in the electric field is observed at the interface between MoS_2_ and AgNPs and it decreases exponentially as one moves away from the interface. In contrast, the electric field intensity remains nearly constant on the surface and inside the film for the bare MoS_2_. Furthermore, the integrated values of the squared E-field over the total MoS_2_ layer are enhanced by 9.6- and 4.5-fold on average for the plasmonic tape structure at the incident wavelengths of 980 nm and 1,550 nm, respectively, compared with those of the bare MoS_2_. Therefore, the localized electric field leads to enhanced NIR absorption in MoS_2_, which is mainly responsible for the increased photocurrent.

In addition to the enhanced NIR absorption in plasmonic tape-MoS_2_, the plasmon excitation may sensitize NIR light, enabling the AgNPs to inject hot carriers into MoS_2_ by absorbing NIR light, because hot electrons can be generated from plasmon decay of Ag nanostructures in the visible and NIR range^33–35^. And, according to previous literatures^21–23,35^, when metals interface with MoS_2_ layers, they may function as localized sources of additional carriers because hot electrons induced from plasmon decay are rapidly transferred to MoS_2_. Therefore, there is a possibility that the generation of hot electrons from the plasmonic tape and the transfer to MoS_2_ contributes in part to the generation of photocurrent.

## Discussion

In this study, we fabricated a multilayer MoS_2_ device attached with a plasmonic tape to detect NIR wavelengths. The NIR photoresponse of the MoS_2_ device was strongly enhanced by directly and simply attaching a Scotch tape with AgNPs onto the MoS_2_ flakes. The plasmonic MoS_2_ device exhibited strongly enhanced photoresponse up to the wavelength of 1,550 nm. We confirmed that the plasmonic-tape-attached MoS_2_ device yielded approximately four times higher photocurrent compared with that of the bare device mainly due to the enhanced NIR absorption, without a noticeable increase in dark current. This plasmonic tape is believed to be applicable to any substrate, including organic substrates that are too weak to be subjected to high-temperature processes.

## Methods

### Preparation of the plasmonic tape

SiO_2_/Si or Si substrates were cleaned via ultrasonication in acetone, washed with ethanol and isopropanol, and subsequently dried. The Trichlorododecylsilane (TCS) treatment of substrates is essential to detach AgNPs easily and simply from substrates using tape. The substrates were treated with UV-ozone for 15 min. They were then immersed into a TCS solution in toluene (99.9%) with 5% volume fraction for 24 h^36,37^. Subsequently, they were cleaned via ultrasonication in toluene and then dried. Subsequently, a 10-nm-thick Ag film was deposited on the substrates using a thermal evaporator. The thin Ag film was then annealed to construct a disordered array of AgNPs on the substrate using a hot plate under the air condition at 220 °C for 1 min. After the annealed substrate was cooled, a 3 M tape was placed on the substrate such that there were no bubbles, and then peeled off. Thus, the plasmonic film was obtained.

### Fabrication of MoS_2_ photodetector

MoS_2_ photodetectors were fabricated by transferring the chemically exfoliated MoS_2_ film onto a polyimide (PI) substrate (Figure S1), and then constructing Au /Cr (100 nm/10 nm) electrodes using a metal shadow mask. The channel length and width of the mask were 20 µm and 200 µm, respectively. Subsequently, a plasmon-enhanced MoS_2_ photodetector was fabricated by attaching the tape with AgNPs carefully onto the MoS_2_ film. The chemically exfoliated MoS_2_ films were fabricated according to a previously reported method^12,24^.

## Supplementary information

Supplementary Information.

## References

[1] Xie C, Yan F (2017). Flexible photodetectors based on novel functional materials. Small.

[2] Xie C, Mak C, Tao XM, Yan F (2017). Photodetectors based on two-dimensional layered materials beyond graphene. Adv. Funct. Mater..

[3] Wang JL (2017). Recent Progress on localized field enhanced two-dimensional material photodetectors from ultraviolet-visible to infrared. Small.

[4] Wang QH, Kalantar-Zadeh K, Kis A, Coleman JN, Strano MS (2012). Electronics and optoelectronics of two-dimensional transition metal dichalcogenides. Nat. Nanotechnol..

[5] Song XF, Hu JL, Zeng HB (2013). Two-dimensional semiconductors: Recent progress and future perspectives. J. Mater. Chem. C.

[6] Choi W (2012). High-detectivity multilayer MoS_2_ phototransistors with spectral response from ultraviolet to infrared. Adv. Mater..

[7] Wang XD (2015). Ultrasensitive and broadband MoS_2_ photodetector driven by ferroelectrics. Adv. Mater..

[8] Xie Y (2017). Ultrabroadband MoS_2_ photodetector with spectral response from 445 to 2717 nm. Adv. Mater..

[9] Wang H, Deng W, Huang LM, Zhang XJ, Jie JS (2016). Precisely patterned growth of ultra-long single-crystalline organic microwire arrays for near-infrared photodetectors. ACS Appl. Mater. Inter..

[10] Joensen P, Frindt RF, Morrison SR (1986). Single-layer MoS_2_. Mater. Res. Bull..

[11] Qiao W (2015). Luminescent monolayer MoS_2_ quantum dots produced by multi-exfoliation based on lithium intercalation. Appl. Surf. Sci..

[12] Eda G (2011). Photoluminescence from chemically exfoliated MoS_2_. Nano Lett..

[13] Park MJ, Yi SG, Kim JH, Yoo KH (2015). Metal-insulator crossover in multilayered MoS_2_. Nanoscale.

[14] Pal B (2017). Chemically exfoliated MoS_2_ layers: Spectroscopic evidence for the semiconducting nature of the dominant trigonal metastable phase. Phys. Rev. B.

[15] Huang JA, Luo LB (2018). Low-dimensional plasmonic photodetectors: Recent progress and future opportunities. Adv. Opt. Mater..

[16] Derkachova A, Kolwas K, Demchenko I (2016). Dielectric function for gold in plasmonics applications: Size dependence of plasmon resonance frequencies and damping rates for nanospheres. Plasmonics.

[17] Zu S (2016). Active Control of plasmon-exciton coupling in MoS_2_-Ag hybrid nanostructures. Adv. Opt. Mater..

[18] Sharma A, Kumar R, Bhattacharyya B, Husale S (2016). Hot electron induced NIR detection in CdS films. Sci. Rep.

[19] Du BW (2017). Plasmonic hot electron tunneling photodetection in vertical Au-graphene hybrid nanostructures. Laser. Photonics Rev..

[20] Venuthurumilli PK, Ye PD, Xu XF (2018). Plasmonic resonance enhanced polarization-sensitive photodetection by black phosphorus in near infrared. ACS Nano.

[21] Hong T (2015). Plasmonic hot electron induced photocurrent response at MoS_2_-Metal junctions. ACS Nano.

[22] Wang WY (2015). Hot electron-based near-infrared photodetection using bilayer MoS_2_. Nano Lett..

[23] Kumar R, Sharma A, Kaur M, Husale S (2017). Pt-nanostrip-Enabled plasmonically enhanced broad spectral photodetection in bilayer MoS_2_. Adv. Opt. Mater..

[24] Park MJ, Park K, Ko H (2018). Near-infrared photodetector achieved by chemically-exfoliated multilayered MoS_2_ flakes. Appl. Surf. Sci..

[25] Wang KP (2014). Broadband ultrafast nonlinear absorption and nonlinear refraction of layered molybdenum dichalcogenide semiconductors. Nanoscale.

[26] Visic B (2011). Optical properties of exfoliated MoS_2_ coaxial nanotubes - analogues of graphene. Nanoscale Res. Lett..

[27] Wang SX (2014). Broadband few-layer MoS_2_ saturable absorbers. Adv. Mater..

[28] Liang LB, Meunier V (2014). First-principles Raman spectra of MoS_2_, WS_2_ and their heterostructures. Nanoscale.

[29] Sandoval SJ, Yang D, Frindt RF, Irwin JC (1991). Raman-study and lattice-dynamics of single molecular layers of MoS_2_. Phys. Rev. B.

[30] Tadi KK, Palve AM, Pal S, Sudeep PM, Narayanan TN (2016). Single step, bulk synthesis of engineered MoS_2_ quantum dots for multifunctional electrocatalysis. Nanotechnology.

[31] Liu CH, Chang YC, Norris TB, Zhong ZH (2014). Graphene photodetectors with ultra-broadband and high responsivity at room temperature. Nat. Nanotechnol..

[32] Yim CY (2014). Investigation of the optical properties of MoS_2_ thin films using spectroscopic ellipsometry. Appl. Phys. Lett..

[33] Haug T, Klemm P, Bange S, Lupton JM (2015). Hot-electron intraband luminescence from single hot spots in noble-metal nanoparticle films. Phys. Rev. Lett..

[34] Lin KQ (2016). Intraband hot-electron photoluminescence from single silver nanorods. ACS Photonics.

[35] Yang XZ (2017). Plasmon-exciton coupling of monolayer MoS_2_-Ag nanoparticles hybrids for surface catalytic reaction. Mater. Today Energy.

[36] Kim J, Takama N, Kim B (2005). Novel microcontact printing technique for multipatterning of self-assembled monolayers. Sensors. Mater..

[37] Zhang Q, Bai RX, Guo T, Meng T (2015). Switchable pickering emulsions stabilized by awakened TiO_2_ nanoparticle emulsifiers using UV/dark actuation. ACS Appl. Mater. Inter..

