# Supplementary material for: Plasmonic-tape-attached multilayered MoS2 film for near-infrared photodetection
Source: Sci Rep. 2020 Jul 9;10:11340. doi: 10.1038/s41598-020-68127-7 (PMC7347569; doi:10.1038/s41598-020-68127-7)
Supplement: Supplementary file 1 — Supplementary Information. [file 41598_2020_68127_MOESM1_ESM.docx]

Supplementary Information

**Plasmonic-tape-attached Multilayered MoS_2_ Film for Near-infrared Photodetection**

*Minji Park ^1^, Gumin Kang ^1^ and Hyungduk Ko^1*^*

Nanophotonics Research Center

Korea Institute of Science and Technology

Hwarangno 14-gil 5, Seongbuk-gu

Seoul 02792, South Korea

Nanophotonics Research Center

Korea Institute of Science and Technology

Hwarangno 14-gil 5, Seongbuk-gu

Seoul 02792, South Korea

*^1^* Nanophotonics Research Center, Korea Institute of Science and Technology, Hwarangno 14-gil 5,
Seongbuk-gu, Seoul 02792, KOREA

*^*^* Corresponding author.
E-mail addresses: kohd94@kist.re.kr


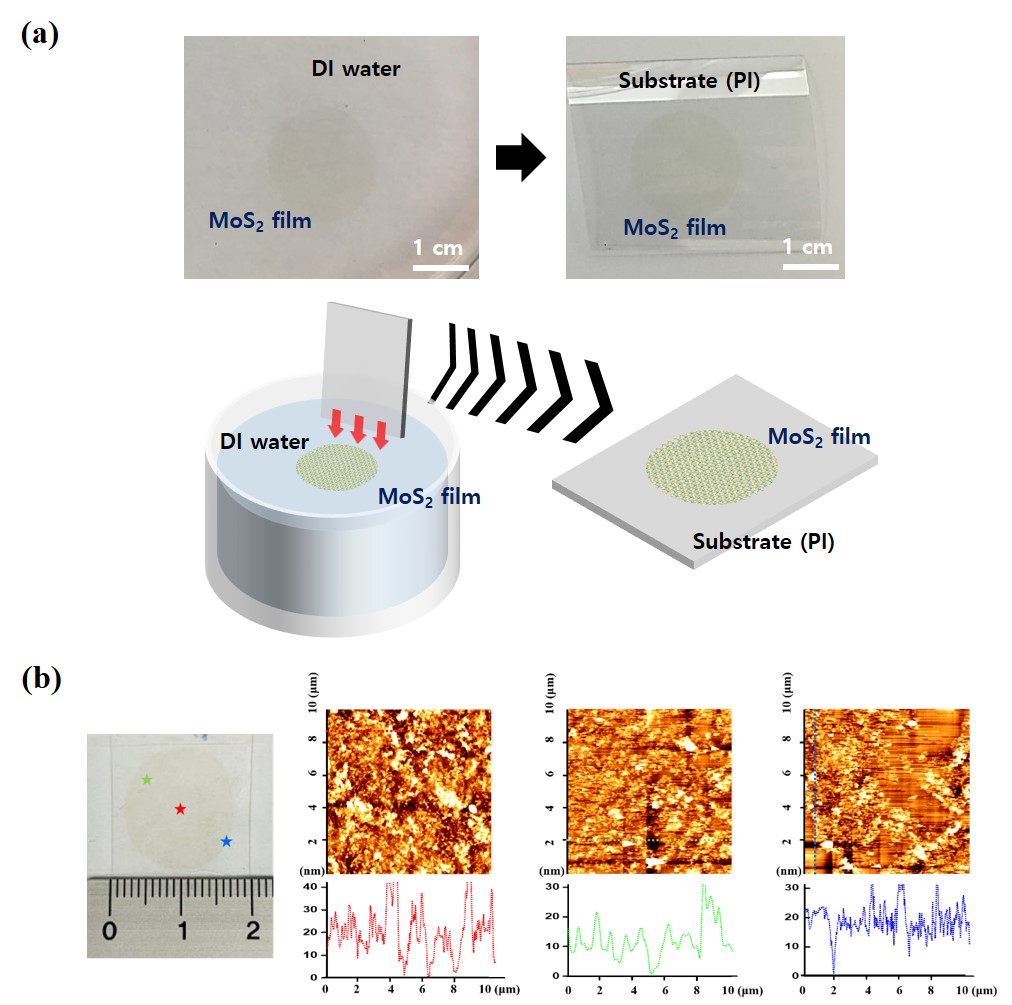


Figure S1. (a) Fabrication process of chemically exfoliated MoS_2_ film on polymer substrate. (b) The thickness distribution of the MoS_2_ films measured using an atomic force microscope (AFM). The thickness of MoS_2_ film was formed uniformly in the centimeter scale.

Figure S2. (a) Schematic of electrode deposition process using a metal shadow mask. (b) Optical image after Au/Cr deposition.


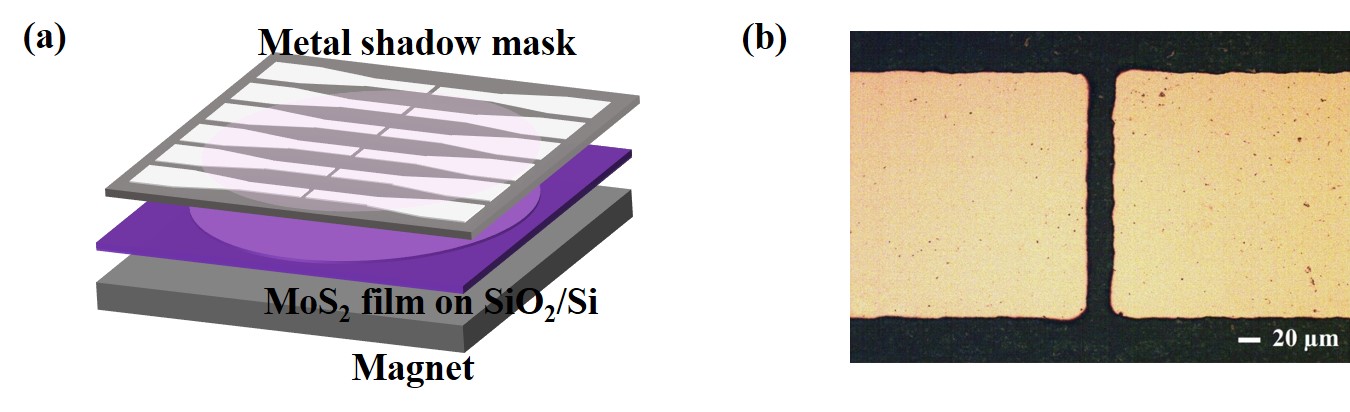

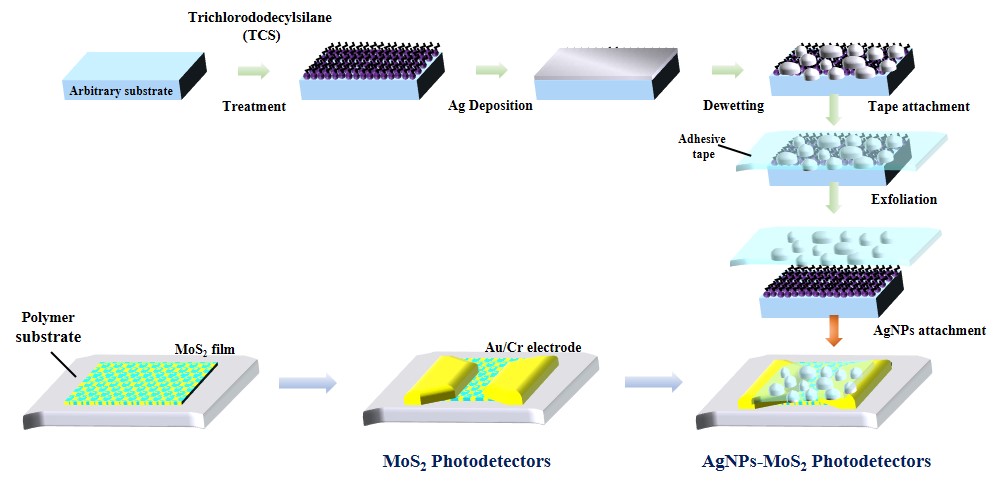


Figure S3. Schematic flow chart of fabrication process for plasmonic-tape-attached MoS_2_ photodetector


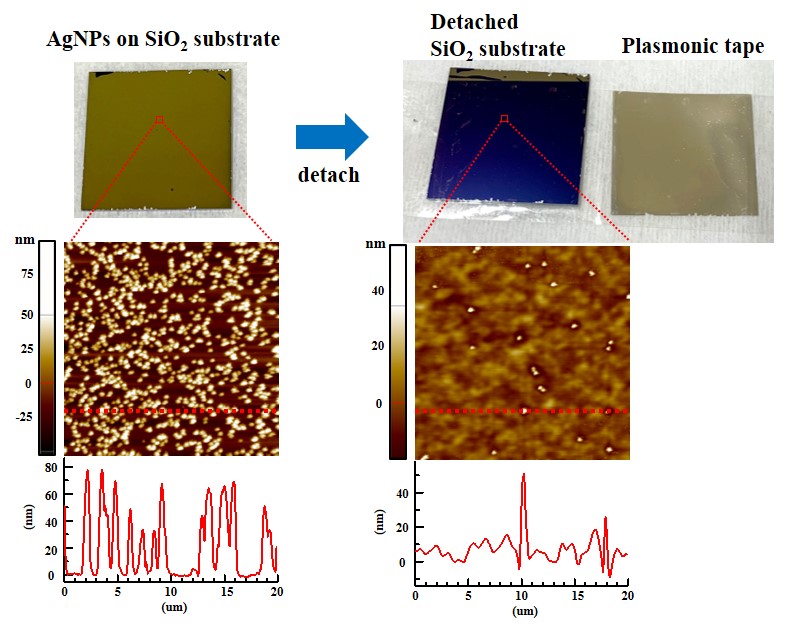


Figure S4. Photographs and AFM images of SiO_2_/Si substrate before and after detaching AgNPs using adhesive tape


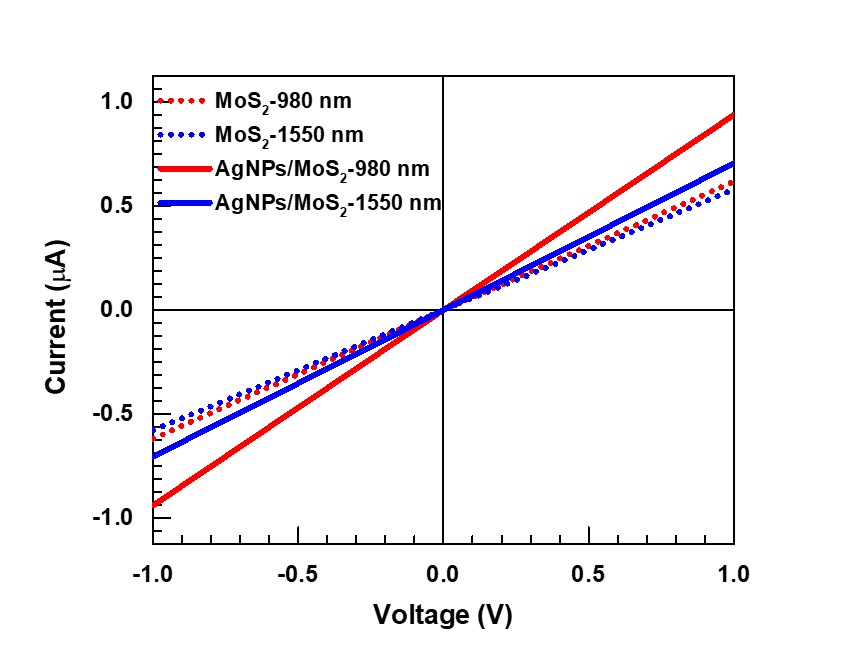


Figure S5. Comparative I*–*V curves of the MoS_2_ photodetectors under the illumination of different wavelengths with the same optical power of ~ 50μW.

Figure S6. Simulated absorption spectra of bare MoS_2_ and plasmonic MoS_2_.


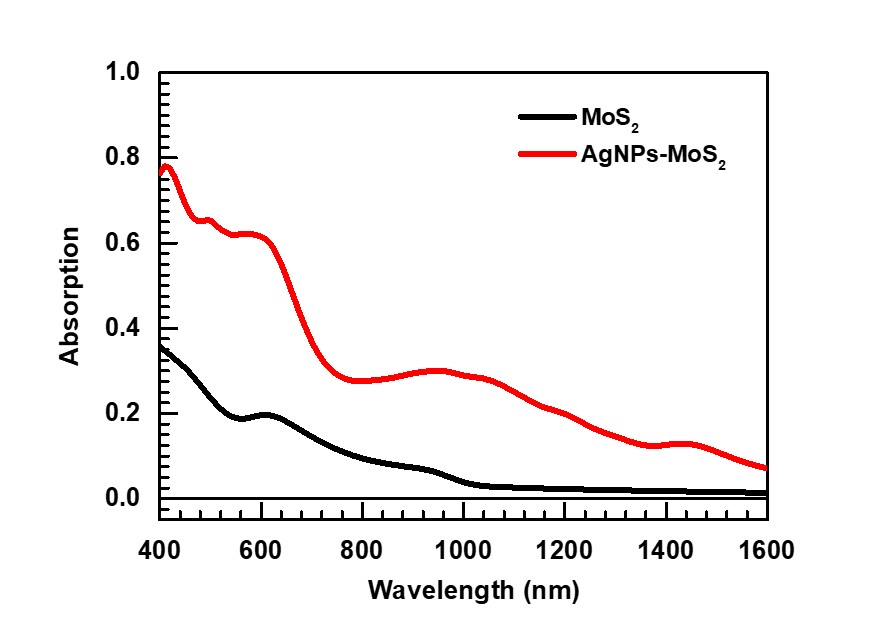


**Table S1.** Plasmonic–assisted 2D material based photodetectors.

| 2D materials based hybrid structures | Preparation of 2D materials | Number of 2D material layers/thickness | λ [nm] | Photocurrent | Responsivity |
| --- | --- | --- | --- | --- | --- |
| Au nanoisland-CdS films ^[18]^ | Chemical bath deposition | – | 400  Halogen lamp  1050 | @1064 nm : 20 pA | @1064 nm : 780 mA/W |
| Au nanoparticles–Graphene ^[19]^ | CVD | monolayer | 600-800 | @710 nm : 12 nA | @710 nm : 0.32nA/μW, at *V_g_* = 1 V |
| Ti/Au bowties–Black phosphorus ^[20]^ | Mechanical exfoliation | 135 nm | 1550 | @1550 nm : 5 μA | @1550 nm : 14.2 mA/W |
| Au electrodes–  MoS_2_ ^[21]^ | Mechanical exfoliation | 6 nm–10 nm | 500 -1050 | @532 nm : 60 nA | - |
| Au nanostructures–MoS_2_ ^[22]^ | Mechanical exfoliation | Bilayer | 532, 1070-1150 | @1150 nm : 5 pA | @1070 nm : 5.2 A/W |
| Pt nanostrips–  MoS_2_ ^[23]^ | Mechanical exfoliation | Bilayer | 325, 532, 980 | @325 nm : 30 nA  @532 nm : 275 nA  @980 nm : 28 nA | @325 nm : 14 A/W, at *V* = 4 V  @532 nm : 312.5 A/W, at *V* = 6 V  @980 nm : 69.2 A/W, at *V* = 1 V |
| Ag nanoparticles-MoS_2_ (Our work) | Chemical exfoliation | ∼20 nm | 980, 1550 | @980 nm : 1 μA  @1550 nm : 0.20 μA | @980 nm : 8.21mA/W, at V = 1 V  @1550 nm : 4 mA/W. at V = 1 V |
